# Preliminary Validation of a Continuum Model for Dimple Patterns on Polyethylene Naphthalate via Ar Ion Beam Sputtering

**DOI:** 10.3390/polym13121932

**Published:** 2021-06-10

**Authors:** Jun-Yeong Yang, Sunghoon Jung, Eun-Yeon Byeon, Hyun Hwi Lee, Do-Geun Kim, Hyo Jung Kim, Ho Won Jang, Seunghun Lee

**Affiliations:** 1 Department of Nano-Bio Convergence, Korea Institute of Materials Science, 797 Changwondae-ro, Changwon 51508, Korea; yjy8184@kims.re.kr (J.-Y.Y.); hypess@kims.re.kr (S.J.); whereks@kims.re.kr (E.-Y.B.); dogeunkim@kims.re.kr (D.-G.K.); 2 Department of Organic Material Science and Engineering, Pusan National University, Busan 46241, Korea; hyojkim@pusan.ac.kr; 3 School of Chemical Engineering, Pusan National University, Busan 46241, Korea; 4 Department of Materials Science and Engineering, Seoul National University, Seoul 08826, Korea; 5 Pohang Accelerator Laboratory, POSTECH, Pohang 790-784, Korea; hhleec@postech.ac.kr; 6 Department of Materials Science and Engineering, Research Institute of Advanced Materials, Seoul National University, Seoul 08826, Korea; hwjang@snu.ac.kr

**Keywords:** ion beam sputtering, polymer, continuum equation

## Abstract

This work reports the self-organization of dimple nanostructures on a polyethylene naphthalate (PEN) surface where an Ar ion beam was irradiated at an ion energy of 600 eV. The peak-to-peak roughness and diameter of dimple nanostructures were 29.1~53.4 nm and 63.4~77.6 nm, respectively. The electron energy loss spectrum at the peaks and troughs of dimples showed similar C=C, C=O, and O=CH bonding statuses. In addition, wide-angle X-ray scattering showed that Ar ion beam irradiation did not induce crystallization of the PEN surface. That meant that the self-organization on the PEN surface could be due to the ion-induced surface instability of the amorphous layer and not due to the partial crystallinity differences of the peaks and valleys. A nonlinear continuum model described surface instability due to Ar ion-induced sputtering. The Kuramoto–Sivashinsky model reproduced the dimple morphologies numerically, which was similar to the experimentally observed dimple patterns. This preliminary validation showed the possibility that the continuum equation used for metal and semiconductor surfaces could be applied to polymer surfaces where ion beam sputtering occurred.

## 1. Introduction

Since the self-organization of nano-dots by ion beam sputtering was introduced by Facsko, diverse nano-structures have been fabricated by ion beam sputtering on semiconductor surfaces [1,2,3]. Regular ripples and dots have been created on the surfaces of Si, InP, and GaSb by noble gas ion beam irradiation [1,4,5,6,7]. Ion-induced surface instability results in self-organization, which has been successfully described by a continuum model including terms of roughening and smoothing [8]. The continuum model accounts for different sputtering yields at peaks and troughs, dependency on the ion’s incident angle, ion-induced effective surface diffusion, thermal diffusion, and surface viscous flow [9]. The similarity between experimentally observed surface morphology and theoretical calculation showed the possibility of exact prediction for self-organization by ion beam sputtering. Nano-dots, ripples, and dimples on polymer substrates also have been observed in surface treatments using ion beam irradiations. M. Goyal reported that a 40 keV oblique argon ion beam resulted in ripple or dot nanostructures on polypropylene (PP) surfaces [10]. We reported that dimple nanostructures were obtained by 1 keV oxygen ion beam treatments on polyethylene terephthalate (PET) surfaces [11]. Polyethylene naphthalate (PEN) surfaces irradiated by an Ar ion beam showed dimple patterns, which resembled the patterns calculated by the continuum equation, especially the Kuramoto–Sivashinsky (KS) model [9]. This similarity implied the possibility of applying the continuum equation to describe the self-organization of polymer surfaces by noble gas ion beam sputtering. In this work, we fabricated dimple patterns on PEN surfaces via 600 eV Ar ion beam irradiation. Surface analysis was performed to find a clue regarding whether ion beam sputtering was the main reaction making the dimple patterns. A nonlinear continuum equation was solved by MATLAB^TM^ software and compared to the dimple patterns obtained by Ar ion beam irradiation.

## 2. Materials and Methods

### 2.1. Materials

A dimple pattern was fabricated on a commercially available PEN film surface by Ar ion beam bombardment. 125 μm PEN film (Dupont Teijin Films, Chester, VA, USA) was prepared by cutting it to a size of 100 × 100 mm^2^. After removing the protective film on the surface, the PEN film was attached to the linear moving stage located in the vacuum chamber.

### 2.2. Ion Beam Treatment

The vacuum chamber was evacuated with a base pressure of 5.0 × 10^−5^ Torr, and then Ar gas was injected into the linear ion source. A gridless ion source was used to generate linear Ar ion beams with a width of 300 mm [11,12]. The PEN samples were treated at a linear moving speed of 10 mm/s. The ion dose per scan was 1.2 × 10^14^ cm^−2^, according to measurement by a Faraday cup. The ion energy distribution function was measured by an ion energy analyzer (Figure S1) [13]. Ar ion beams were irradiated under normal incidence conditions.

### 2.3. Field-Emission Scanning Electron Microscopy

The PEN surface was observed using field-emission scanning electron microscopy (FE-SEM; JSM 6700F, JEOL, Tokyo, Japan) in secondary electron (SE) mode. The accelerating voltage was maintained at 5 kV.

### 2.4. Atomic Force Microscopy

The morphology of the dimple nanostructures was measured by atomic force microscopy (AFM, NX10, Park Systems, Suwon, Korea) in non-contact mode.

### 2.5. Field-Emission Transmission Electron Microscopy and the Electron Energy Loss Spectrum

A localized cross-linking on the dimple nanostructures was verified via cross-sectional field-emission transmission electron microscopy (FE-TEM, JEM-ARM, JEOL, Tokyo, Japan) with electron energy loss spectroscopy (EELS, JEOL, Tokyo, Japan). The FE-TEM measurements were performed at an accelerating voltage of 200 kV. The cross-sectional specimens of FE-TEM were prepared using a liquid metal ion source (Ga^+^) equipped with a focused ion beam with a coarse milling current (4 nA) and a fine milling current (20 pA) at 30 kV. The 90 nm–thick PEN sample was prepared with a focused ion beam.

## 3. Results and Discussion

**Figure 1:**
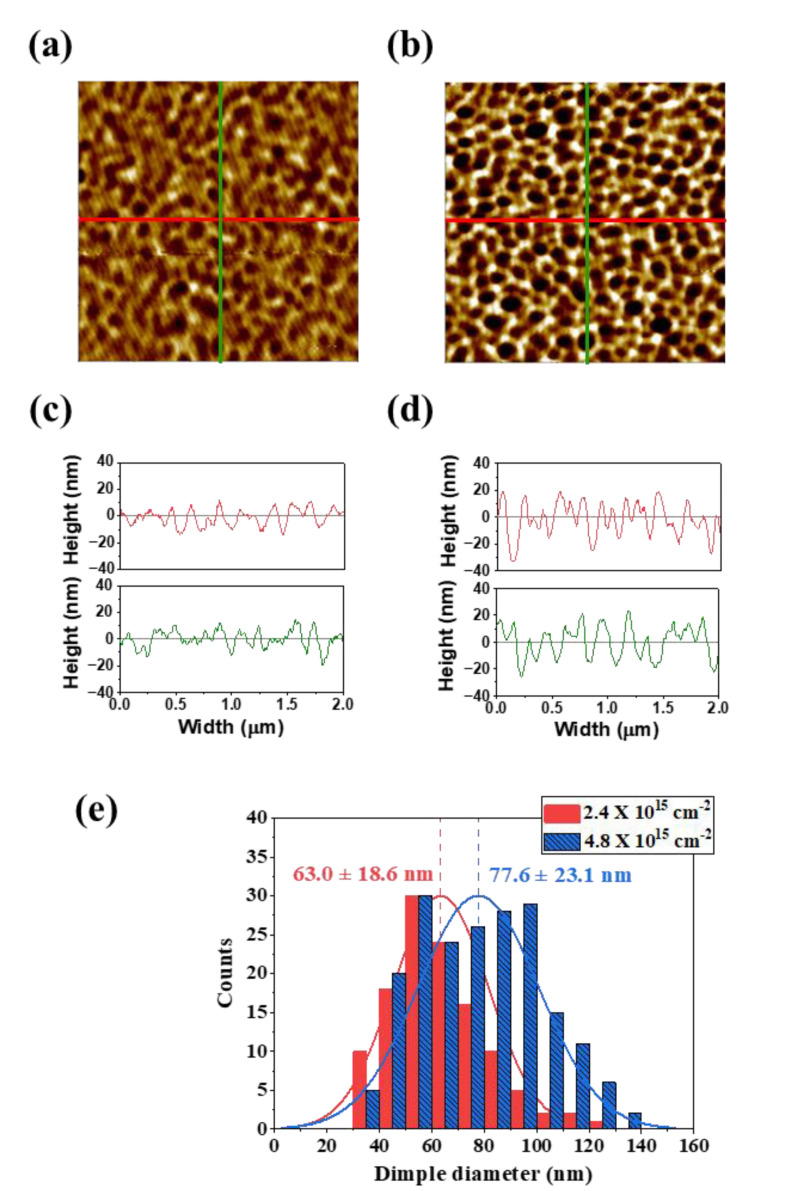
AFM images (area: 2 μm × 2 μm) of dimple patterns on a PEN surface by 600 eV Ar ion beam bombardment with ion doses of (**a**) 2.4 × 10^15^ cm^−2^ and (**b**) 4.8 × 10^15^ cm^−2^. *X*-axis and *Y*-axis line profiles: (**c**) 2.4 × 10^15^ cm^−2^, (**d**) 4.8 × 10^15^ cm^−2^. (**e**) Distribution of dimple diameter in the 2 μm × 2 μm area.

Figure 1 shows surface morphologies and profiles. Figure 1a,b show AFM images of PEN surfaces treated by 600 eV Ar ion beam bombardment with ion doses of 2.4 × 10^15^ cm^−2^ and 4.8 × 10^15^ cm^−2^, respectively. As the ion dose was increased, the irregular dimple patterns were formed clearly. In Figure 1c,d, the peak-to-peak roughness (R_z_) was increased from 29.1 ± 4.7 nm and 53.4 ± 8.4 nm as the ion dose was increased. In Figure 1e, a scanning probe image processor (SPIP^TM^, Image Metrology, Diplomvej, Denmark) showed that dimple diameter was increased from 63.4 ± 18.6 nm to 77.6 ± 23.1 nm as the ion dose was increased (Figure S2). In addition, the aspect ratio (R_z_/diameter) of dimples were increased from 0.45 to 0.68.

Self-organization by ion beam irradiation has been explained by two mechanisms. One is the semi-crystallinity of cross-linked polymers. The sputtering yield of a semi-crystalline polymer is lower than that of the amorphous phase [14]. For instance, He ion bombardment on polytetrafluoroethylene surfaces left the backbones of the crystallized polymer chains, which formed worm-like structures [15]. Another possibility is surface instability induced by ion bombardments. Ion-induced surface instability results in roughening and smoothing of the amorphous layer [2]. The self-organization of ordered morphologies by ion beam sputtering is independent from the orientation of surface materials because self-organization by surface instability occurs in the amorphous layer formed by ion beam irradiation [2]. If the polymer surface is in the amorphous phase during ion beam sputtering, ion-induced surface instability could explain the pattern formation.

**Figure 2:**
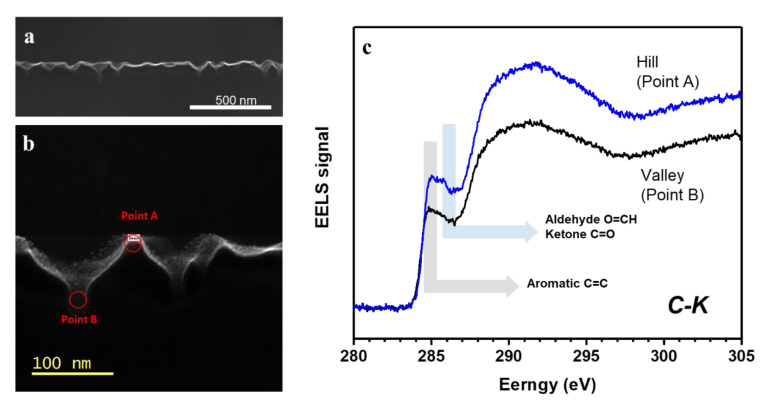
(**a**) Cross-sectional SEM image of dimple pattern, (**b**) the points of EELS measurement at a peak and trough. (**c**) EELS at the selected peak and trough.

In Figure 2, FE-TEM and EELS show the cross-sectional images and the chemical bond statuses at the valleys and hills of the dimple structures. The EELS was measured at the peak (point A in Figure 2a) and trough (point B in Figure 2b). The EELS signal showed aromatic C=C (285 eV), ketone C=O (286.3–286.8 eV), and aldehyde O=CH (286.3–286.8 eV) functional groups, which existed similarly at the peak and trough [16]. If the partially distributed crystalline chains induced the dimple patterns, the binding status at the peak position would differ from that at the trough region. This revealed that the patterning mechanism was not partial semi-crystallization. Wide angle X-ray scattering (WAXS) showed that semi-crystallization was not induced by Ar ion beam irradiation (Figure S3). The EELS and WAXS results mean that the self-organization on the PEN samples could be due to the ion-induced surface instability of the amorphous layer.

**Figure 3:**
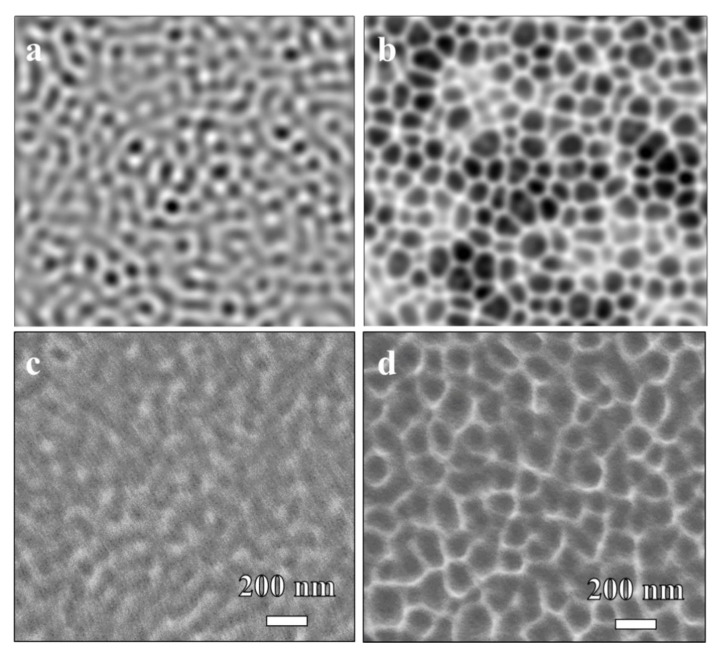
Simulation results by iterating the DKS equation: (**a**) ion flux = 4.83 × 10^13^ cm^−2^s^−1^, time = 50 s for 20 scans (**b**) ion flux = 4.83 × 10^13^ cm^−2^s^−1^, time = 100 s for 40 scans. Experimentally obtained dimple patterns on the PEN surface: (**c**) 20 scans, (**d**) 40 scans.

The KS model could explain the self-organization of dimple structures on the PEN surface by ion-induced surface instability. The mechanism of pattern formation by ion bombardment has been extensively investigated using a continuum equation [8,9]. The KS equation describes the morphology evolution during an ion bombardment. The KS equation is given by

(1) $$\frac{\partial h}{\partial t}=\nu \nabla^{2}h-D\nabla^{4}h-K\nabla^{4}h-\lambda {\left(\nabla h\right)}^{2}+\eta$$

where *h* is the height of the ion-bombarded surface as a function of time *t*, *ν* is the effective surface tension generated by the erosion process or viscous flow due to surface stress, *D* is the diffusion coefficient from ion-induced diffusion, *K* is a thermal diffusion coefficient, *λ* describes the tilt-dependent sputtering yield, and *η* is Gaussian white noise resulting from the stochastic nature of the erosion process. We tried to reproduce the dimple structures on PEN surfaces by Ar ion beam irradiations using the KS model. Numerical simulation was executed on an equally spaced, 2-dimensional 200 × 200 mesh by integration of Equation (1) using a standard discretization method with periodic boundary conditions. The integration began from a random surface with a height randomly distributed from 0 to 0.1 with spatial step d*x* = 0.5 nm, time step d*t* = 0.002 s, *ν* = −0.0553 nm^2^/s, *λ* = −0.177 nm/s, *D* = 0.0138 nm^4^/s, and *K* = 0. The coefficients were calculated by ion species (Ar), polymer density (1.36 g/cm^−3^), ion energy (600 eV), and a definition of coefficients from previous work [8,17]. The sputtering yield was calculated by SRIM code [17]. The sign of the nonlinear term *λ* determined dot or dimple structures. A positive *λ* induced dot formation and a negative *λ* described dimple formation [2,8]. In this normal incident condition, Equation (1) yielded an isotropic, partial differential equation with a negative nonlinear *λ* term, which implied dimple formation. Figure 3 compares simulation results using Equation (1) with FE-SEM images of experimentally fabricated samples. The images reveal a similarity of surface morphology between the simulation and the experiment. In the simulation, the disconnected patterns developed after 50 s, which corresponded to 20 scans (Figure 3a). After 100 s (40 scans), dimple patterns formed (Figure 3b). As experimental bombardment proceeded (Figure 3c), a partially disconnected network structure was observed after 20 scans. For longer irradiation times, the pattern formed after 40 scans (Figure 3d). The spatiotemporal pattern predicted by Equation (1) showed good correlation with experimental observations.

The successful prediction of self-organization means that ion beam irradiation on the PEN surface mainly results in surface sputtering. The continuum equation is based on the sputtering reaction on the ion beam irradiated surface. Ion irradiation on the polymer surface simultaneously induces cross-linking and sputtering. Ion energy transfer by nuclear collision affects the ratio of cross-linking to sputtering. If surface atoms receive sufficient energy to break surface binding with energetic recoils and ions, surface displacement induces sputtering. Energy transfer by recoils and ions depends on the atomic number density of the target polymer. The concept of displacement per atom (DPA), which is proportional to the energy transfer by the nuclear stopping reaction, could be a quantitative value for evaluating the degree of displacement [18]. PEN density (1.36 g/cm^3^) was sufficient to induce multiple atomic displacements of polymer atoms at the surface. The 600 eV Ar collision on the PEN surface showed a DPA of 4 to 8, a value sufficient for inducing frequent sputtering by ion bombardments. Thus, the continuum model based on sputtering reactions showed similar surface morphology to an Ar ion beam-irradiated PEN surface.

## 4. Conclusions

The comparison of the KS model and the dimple pattern showed the validity of applying the continuum model to the self-organization of a polymer surface using ion beam irradiation. The continuum model based on sputtering reactions could be applied to the limited polymer material, which has a density that induces sufficient surface displacement and sputtering by ion beam irradiation.

The discrepancy between the simulation and the experiment is due to a limitation in explaining the exact ion-induced sputtering yield of the polymer substrate that consists of several atoms such as carbon, hydrogen, and oxygen. The SRIM code supplied a statistically averaged sputtering yield for the polymer substrate.

This approach could be useful to fabricate nanostructures applied to sensors such as surface-enhanced Raman spectroscopy, which uses the enhancement of local electric fields wherein the surface morphology has a several nm gap [19,20]. The dimple patterns could be applied to form hot spots on polymer surfaces using a simple, top-down process.

## Data Availability

Not applicable.

## Supplementary Materials

The following are available online at https://www.mdpi.com/article/10.3390/polym13121932/s1, **Figure S1.** Linear ion beam irradiation on a moving substrate (speed: 10 mm/s). Ion current density and ion energy distribution function were measured by Faraday cup and retarding potential ion energy analyzer, respectively; **Figure S2.** Diameter measurement of dimple structure using SPIP^TM^ software, (**a**) ion dose: 2.4 × 10^15^ cm^-2^, (**b**) ion dose: 4.8 × 10^15^ cm^−2^; **Figure S3.** Spectrum of wide angle X-ray scattering, (**a**) as-received PEN, (**b**) 600 eV Ar ion-beam irradiated PEN.
