# Supplementary material for: Preliminary Validation of a Continuum Model for Dimple Patterns on Polyethylene Naphthalate via Ar Ion Beam Sputtering
Source: Polymers (Basel). 2021 Jun 10;13(12):1932. doi: 10.3390/polym13121932 (PMC8230451; doi:10.3390/polym13121932)
Supplement: Supplementary file 1 [file polymers-13-01932-s001.zip › polymers-1233684-supplementary.pdf]

polymers

MDPI

Supplementary Materials

Preliminary Validation of a Continuum Model for Dimple Patterns on Polyethylene Naphthalate via Ar Ion Beam Sputtering

Jun-Yeong Yang [1,2,3,+], Sunghoon Jung [1,4,+], Eun-Yeon Byeon [1], Hyun Hwi Lee [5], Do-Geun Kim [1], Hyo Jung Kim [2,3], Ho Won Jang [6] and Seunghun Lee [1,*]

[1] Department of Nano-Bio Convergence, Korea Institute of Materials Science, 797 Changwondae-ro, Changwon 51508, Korea; yjy8184@kims.re.kr (J.-Y.Y.); hypess@kims.re.kr (S.J.); whereks@kims.re.kr (E.-Y.B.); dogeunkim@kims.re.kr (D.-G.K.)

[2] Department of Organic Material Science and Engineering, Pusan National University, Busan 46241, Korea; hyojkim@pusan.ac.kr

[3] School of Chemical Engineering, Pusan National University, Busan 46241, Korea

[4] Department of Materials Science and Engineering, Seoul National University, Seoul 08826, Korea

[5] Pohang Accelerator Laboratory, POSTECH, Pohang 790-784, Korea; hhleec@postech.ac.kr

[6] Department of Materials Science and Engineering, Research Institute of Advanced Materials, Seoul National University, Seoul 08826, Korea; hwjang@snu.ac.kr

* Correspondence: seunghun@kims.re.kr

\+ These authors are contributed equally.

Citation: Yang, J.-Y.; Jung, S.; Byeon, E.-Y.; Lee, H.H.; Kim, D.-G.; Kim, H.J.; Jang, H.W.; Lee, S. Preliminary Validation of Continuum Model for Dimple Pattern on Polyethylene Naphthalate via Ar Ion Beam Sputtering. *Polymers* **2021**, *13*, 1932. https://doi.org/10.3390/polym13121932

Academic Editor: Choon-Sang Park

Received: 8 May 2021
Accepted: 8 June 2021
Published: 10 June 2021

Publisher's Note: MDPI stays neutral with regard to jurisdictional claims in published maps and institutional affiliations.

Copyright: © 2021 by the authors. Licensee MDPI, Basel, Switzerland. This article is an open access article distributed under the terms and conditions of the Creative Commons Attribution (CC BY) license (http://creativecommons.org/licenses/by/4.0/).

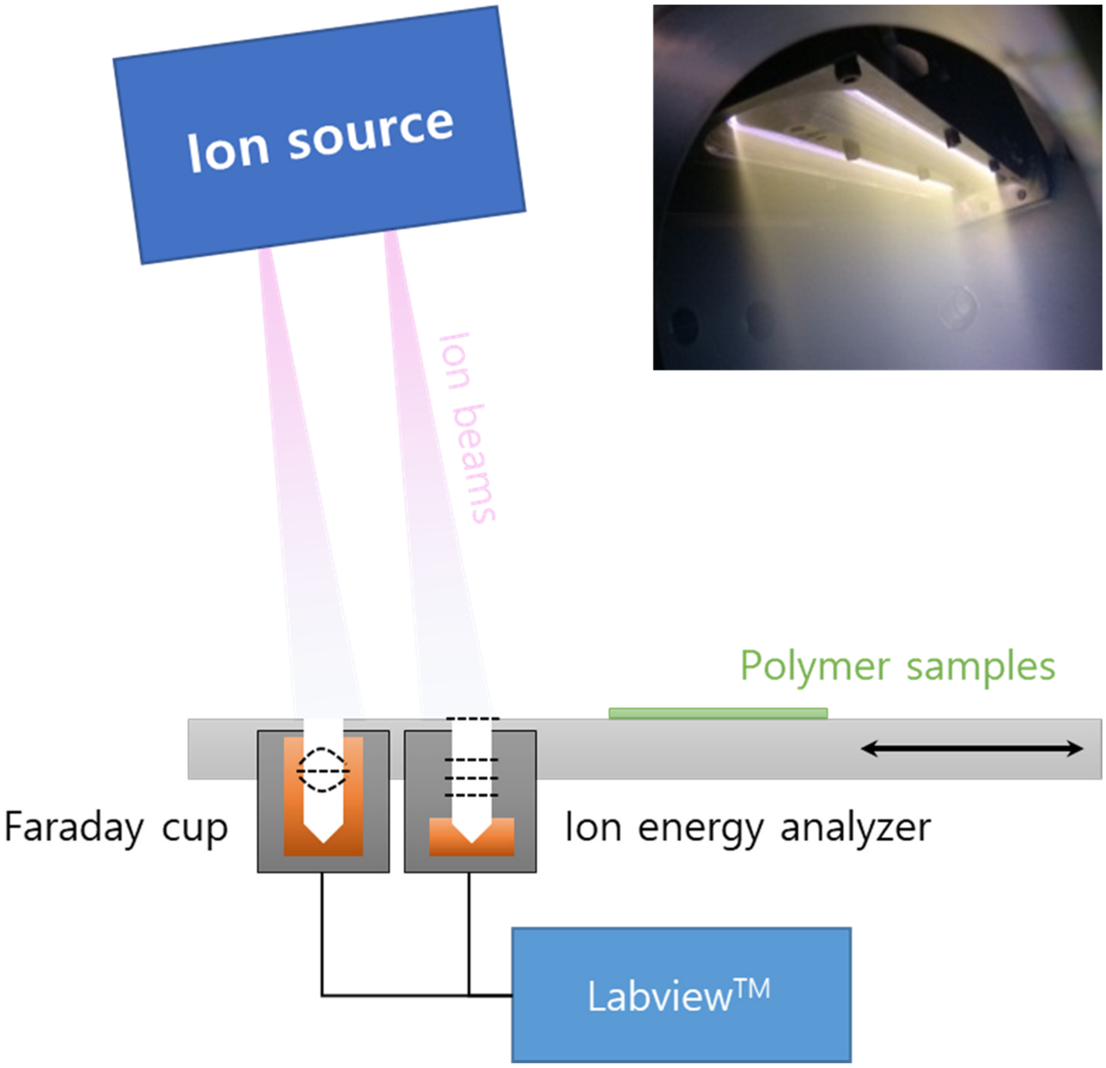

Figure S1. Linear ion beam irradiation on a moving substrate (speed: 10 mm/s). Ion current density and ion energy distribution function were measured by Faraday cup and retarding potential ion energy analyzer, respectively.

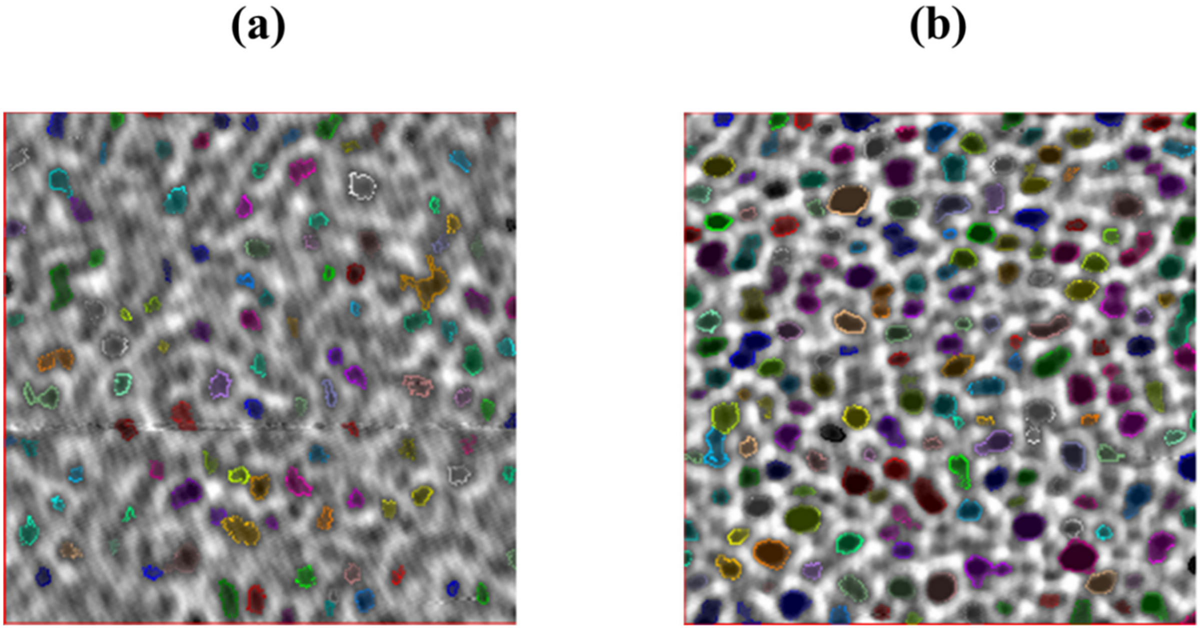

Figure S2. Diameter measurement of dimple structure using SPIP™ software, (**a**) ion dose: 2.4 × 10^{15} cm^{-2}, (**b**) ion dose: 4.8 × 10^{15} cm^{-2}.

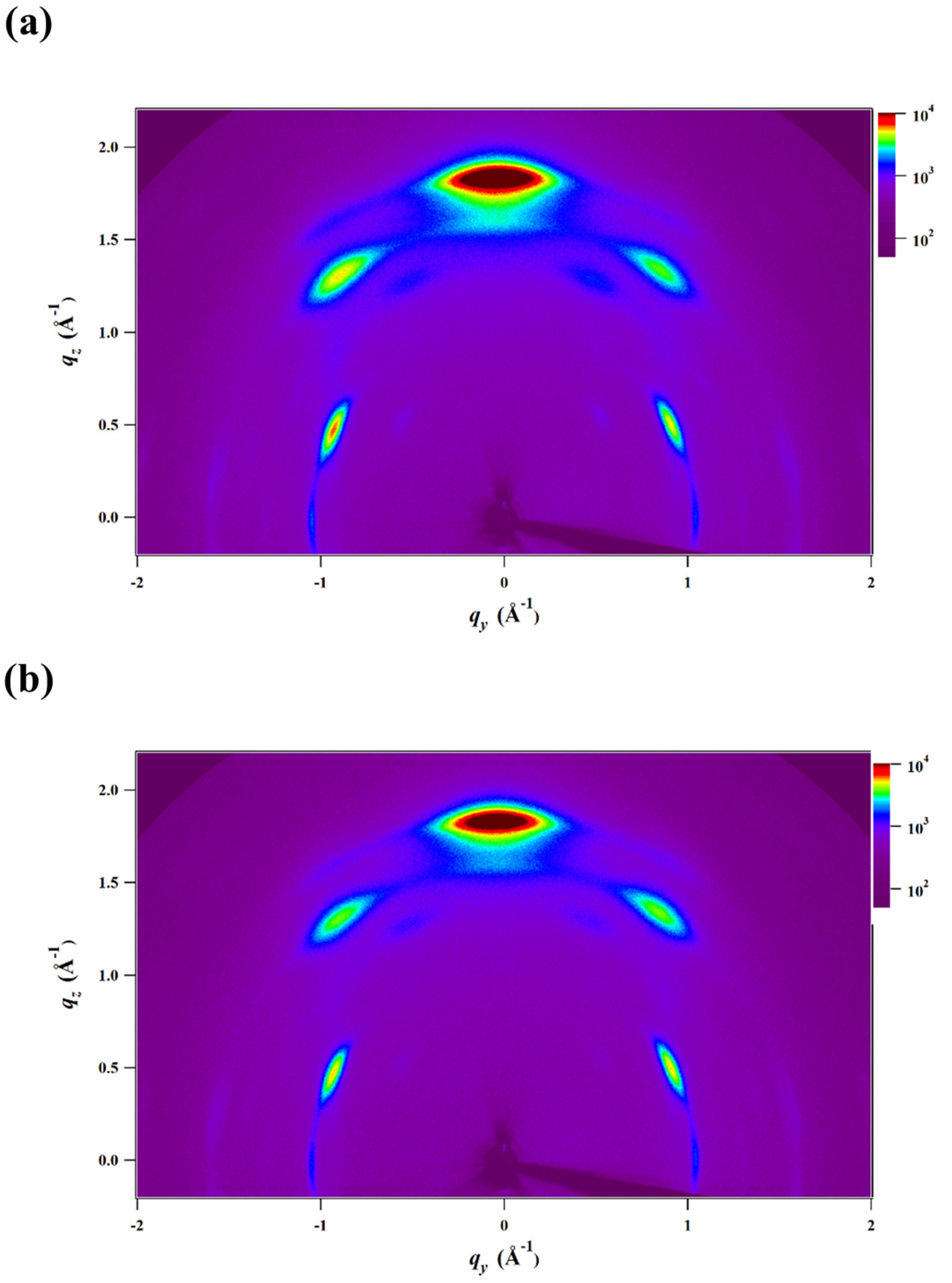

Figure S3. Spectrum of wide angle X-ray scattering, (**a**) as-received PEN, (**b**) 600 eV Ar ion-beam irradiated PEN.
